# Preliminary development of a scale to measure stigma relating to sexually transmitted infections among women in a high risk neighbourhood

**DOI:** 10.1186/1472-6874-8-21

**Published:** 2008-11-20

**Authors:** Melanie LA Rusch, Jean A Shoveller, Susan Burgess, Karen Stancer, David M Patrick, Mark W Tyndall

**Affiliations:** 1 Division of International Health and Cross Cultural Medicine, University of California San Diego, La Jolla, USA; 2 Department of Health Care and Epidemiology, University of British Columbia, Vancouver, Canada; 3 Department of Family Practice, University of British Columbia, Vancouver, Canada; 4 Downtown Community Health Centre, Vancouver Coastal Health, Vancouver, Canada; 5 British Columbia Centre for Disease Control, Vancouver, Canada; 6 British Columbia Centre for Excellence in HIV/AIDS, Vancouver, Canada; 7 Department of Medicine, University of British Columbia, Vancouver, Canada

## Abstract

**Background:**

As stigma is a socially constructed concept, it would follow that stigma related to sexual behaviours and sexually transmitted infections would carry with it many of the gender-based morals that are entrenched in social constructs of sexuality. In many societies, women tend to be judged more harshly with respect to sexual morals, and would therefore have a different experience of stigma related to sexual behaviours as compared to men. While a variety of stigma scales exist for sexually transmitted infections (STIs) in general; none incorporate these female-specific aspects. The objective of this study was to develop a scale to measure the unique experience of STI-related stigma among women.

**Methods:**

A pool of items was identified from qualitative and quantitative literature on sexual behaviour and STIs among women. Women attending a social evening program at a local community health clinic in a low-income neighbourhood with high prevalence of substance use were passively recruited to take part in a cross-sectional structured interview, including questions on sexual behaviour, sexual health and STI-related stigma. Exploratory factor analysis was used to identify stigma scales, and descriptive statistics were used to assess the associations of demographics, sexual and drug-related risk behaviours with the emerging scales.

**Results:**

Three scales emerged from exploratory factor analysis – female-specific moral stigma, social stigma (judgement by others) and internal stigma (self-judgement) – with alpha co-efficients of 0.737, 0.705 and 0.729, respectively. In this population of women, internal stigma and social stigma carried higher scores than female-specific moral stigma. Aboriginal ethnicity was associated with higher internal and female-specific moral stigma scores, while older age (>30 years) was associated with higher female-specific moral stigma scores.

**Conclusion:**

Descriptive statistics indicated an important influence of culture and age on specific types of stigma. Quantitative researchers examining STI-stigma should consider incorporating these female-specific factors in order to tailor scales for women.

## Background

Stigma has long been a part of our social existence, with the original Greek translation referring to a physical sign exposing a moral imperfection[1]. While in today's society the physical mark need not be present, the moral associations have remained intact. The topic of sexually transmitted infections (STIs) presents a good example of the dynamic and socially fluid nature of stigma, as opposed to the stationary, objectified definition it is sometimes given [2]. In relation to the categories of stigma outlined in Goffman's (1963) foundational work, STIs could be argued to cross all three – stigma of the body, of moral character and of tribe[1,3]. In addition, for any one individual, STIs could also blur the boundaries of the discredited – one who is overtly stigmatized – and the discreditable – one who may be able to conceal their stigmatizing feature – depending on the nature of social interaction at any particular time[1,4].

Sexual morals have typically had a gender imbalance, leading to a stronger social stigmatization of women. Many societies and cultures view promiscuity among men favourably (e.g., as a measure of virility or status), while promiscuity among women is viewed as undesirable and immoral [3,5,6]. In the late 19^th ^and early 20th centuries, the social and medical standpoints on the spread and prevention of STIs were influenced strongly by these gender stereotypes. For example, in World War I, STI prevention flyers were used to warn soldiers away from the 'dirty' women who would infect them with STIs, which they might then pass on to their 'good' wives [7]. Prior to available treatment, the impact of STI sequelae was so great among soldiers, some states enacted laws against 'promiscuity', and many single women were arrested or detained for such things as being out at a bar or club on their own [7]. Today's views on sexual behaviours and STIs may not be as overtly imbalanced, but there remains an underlying gender bias in the stereotypes and the meanings associated with STIs, resulting in different stigma experiences and generally higher negative impacts among women [8,9].

The continued impact of the good/bad dichotomy on women's perceptions of STIs is captured in the qualitative work of Nack (2002)[3]. In her work, discourse evolved around sexual behaviour norms and behaviour that was deemed appropriate for women evolved around the moral division of respectable or 'good girls' and disreputable or 'bad girls', leading to the development of the idea of 'tribes of womanhood'. Membership in the 'good girl tribe' or morally-correct category, whether through actual behaviour, avoidance of STI or concealment of behaviours or diagnoses, was precarious, while membership in the 'bad girl tribe', was easy to gain and often thought of as irreversible.

In addition to the potential psychological harms an individual might deal with when faced with a positive STI test result, there may also be an impact on testing and treatment behaviours at the population level. In part, this may be explained by the additional STI-related stigma that can arise within health care settings (e.g., patients must reveal the relevant details of their sexual behaviour in order to seek help from caregivers, thereby risking becoming discredited; patients may also fear being discredited to other clinic staff who may have access to their charts). Patient comfort, appropriate staff communication, confidentiality and respect for the feelings of the women have been identified as key stigma-related factors that need to be addressed in the provision of STI services[10]. A similar study in the southern U.S. outlined four important concepts of stigma that surfaced from qualitative focus groups, including religious ideation of health care workers affecting their views of 'promiscuous' women, privacy fears among men, racial attitudes and stigma transference or fear of being labelled [11]. Thus, the ability to present safely and comfortably in a clinic setting can be disturbed by actual or perceived discriminating attitudes of the other clinic attendees, the doctors and nurses, or other clinic staff.

Stigma scales exist for general disabilities, mental health, and more recently for HIV/AIDS [12-15]. There are, however, few scales that examine stigma in relation to STIs (notably, Fortenberry's stigma and shame scales[16,17]) and none that incorporate female stereotypes related to sexual morals, as well as the perceptions of both the community in general and health care professionals in particular. The purpose of this study was to develop a stigma scale specific to women, which encompassed a broader range of the stigma experience associated with sexuality and STIs. The present paper outlines the preliminary development of such a scale, and assesses the demographic and behavioural characteristics associated with the resulting scales.

## Methods

An interviewer-administered structured survey was carried out among 126 women attending a weekly program exclusive to women (including transgendered individuals) held at a local community health clinic in Vancouver's Downtown Eastside. This region is disproportionately impacted by issues including substance use, mental illness, homelessness and poverty. Compared to the rest of the province, census data from 2006 indicated higher levels of income assistance (4.1% vs. 0.6%), higher levels of low income households (23.9% vs. 12% of families earning < $20,000 CDN), and inferior health outcomes (life expectancy at birth 75 vs 81 years)[18]. Due to the concentration of injection drug use and sex work in this high risk neighbourhood, the region also exhibits high prevalence of HIV and other STIs[19-21].

The women's program is open to all women, and sees anywhere from 20 to 60 women in one evening (for more detailed information on the program and population, see Rusch, et al. 2008 [22]). The weekly, three-hour program offers women a safe place to access food and health care, as well as to socialize with other women, and take part in various activities including free haircuts, foot baths, art projects, and movie nights. There is also access to doctors and nurses, counselling services and massage therapy. The program is advertised through fliers at the clinic and other community organizations frequented by women. Women were passively recruited through an announcement at the start of each evening inviting women to take part in the study. Although there was no overt advertising for the study, snowball sampling through word-of-mouth was used to recruit women, including those women who may not have been accessing the evening program on a regular basis.

The 27-item structured interview was used to gather data to describe the socio-demographic characteristics of the study participants, their use of and contact with services available in their community (including their contact with outreach programs, outreach workers and street nurses), as well as their self-reported patterns of sexual behaviour and drug use. We also asked questions about their use of sexual health care services such as annual pap smears and testing or treatment for STIs. The purpose of the larger study was to determine the characteristics and risk levels of the women attending the program in order to help inform program planners and to help tailor outreach initiatives for those women missing from the demographic, as well as to assess potential barriers to sexual health care among this population of women.

Participants were given a copy of the consent form, and study coordinators read through the details of participation before asking for their consent. Regular clinic staff and doctors were not actively involved in the recruitment or interviewing, and the consent included a statement reassuring participants that if they chose not to participate, there would be no consequences to their involvement with the clinic or the women's night program, nor their normal receipt of care at the clinic. As part of the larger study, participants had the option of providing a urine sample for Chlamydia and gonorrhoea screening; however, this was not a requirement for taking part in the study. The clinic provided follow-up care for participants testing positive. Participants received $10 remuneration for completing the structured interview. This study was approved by the University of British Columbia Behavioural Ethics Review Board.

While transgendered individuals were not excluded from participating in the study, they were not included in the present analysis, as STI-stigma perceptions may be very different in this population and there were insufficient numbers (N = 4) to allow for comparison.

An 18-item pool was created building on Goffman's (1963) basic three categories, drawing on previous constructs from a general STI stigma and shame scale by Fortenberry et al (2002), and incorporating the idea of the tribes of womanhood introduced by Nack (2002)[1,3,17]. In addition, based on other discourse around stigma and sexual health care seeking behaviours, items relating to Goffman's category of 'moral' stigma included both internal feelings of guilt or shame, as well as the perceived views of others in the community, including health care workers and intimate partners [10,23]. Items were also included to encompass concerns over discretion, confidentiality and gossip. While the items were developed and selected based on the pre-existing theories and literature mentioned above, the analysis was exploratory rather than theory-driven and therefore did not presuppose any categories or groupings of the items.

Items were assessed for endorsement using a discrimination index, and for internal consistency within subgroups using Pearson's correlations. Problematic items were highlighted and taken into account during exploratory factor analysis.

Exploratory principal component factor analysis was done using promax (oblique) rotation to explore the categories present in the responses. An iterative process was used, discarding one item at a time based on factor loadings as well as on discretion and internal consistency results, where applicable. From the original 18 items, four were discarded due to poor discrimination or poor factor loading. For each of the remaining 14 items, non-responders were compared to responders in order to assess variability.

For the three resulting scales (female-specific moral stigma, social stigma and internal stigma), item-total correlation and alpha scores were calculated. Means and standard deviations are also presented. Associations of the three final scales with demographic and behavioural characteristics were assessed using the Wilcoxon rank-sum test.

## Results

Demographics of the 126 participants are shown in Table 1. The median age was 42 years, approximately 40% identified as Aboriginal, Inuit or Métis, and approximately 40% reported having completed high school. Unemployment was high (95%), as was substance use (injection drugs: 40%, non-injection drugs: 80%). Thirty-eight percent of the women were currently involved in sex trade.

**Table 1:** Demographics of 126 participants attending a women's program in a community clinic located in a low-income, high risk neighbourhood

	**Total** **(N = 126)**
**Gender**	
Female	96.8 (122)
Transgender	3.2 (4)
	
**Median age (IQR)**	42 (36, 49)
	
**Ethnicity**	
White	52.4 (66)
Aboriginal	39.7 (51)
	
**Education**	
<High school	59.5 (75)
High school	40.5 (51)
	
**Employment**	
Any	5.6 (7)
None	94.4 (119)
	
**Drug Use**	
Injection	39.7 (48)
Non-injection	81.8 (99)
Alcohol	41.6 (52)
	
**Sexual partners (past 6 mos)**	
Regular partner	57.9 (73)
Casual partner	20.6 (26)
>1 partner	45.6 (57)
	
**Commercial Sex Work status**	
Non-	30.2 (38)
Former	26.2 (33)
Current	38.1 (48)
	
**Attendance at Weekly Women's Program**	
Never	23.8 (30)
Sometimes/Regularly	76.2 (96)

Table 2 outlines the STI-related stigma items included in the structured interview. Items included feeling dirty, feeling violated, knowing (and conversely, not knowing) that an STI was present, being able to hide an STI from others, feeling guilty, feeling embarrassed, perceiving those with an STI as having low intelligence, bad character and specifically, bad character as judged by clinic staff. Four items incorporated concepts introduced by Nack (2000, 2002) elucidating what women perceived to be the "type of woman" who gets an STI: 1) being 'damaged goods', 2) being promiscuous, 3) being at fault as "women should 'know better"' and 4) being at fault for not being "careful enough"[3,4]. Lastly, four items were included to encompass discretion of clinic setting and clinic staff, concern regarding community gossip, and fear of repercussions from partner disclosure. It was felt that including items regarding discretion and disclosure would build on the concept of social judgement, potentially allowing a subtle distinction between women who perceived others to be judgemental and women who were concerned about being judged, or becoming, even in a hypothetical setting, discredited.

**Table 2:** Items developed for STI-related stigma scale, measured on a 10-point scale (1 = strongly disagree, 10 = strongly agree)

***If you had an STI, you would feel:***	**Mean (std)**	**Median (IQR)**
1. Dirty	7.11 (3.42)	8 (5, 10)
2. Violated	7.58 (3.24)	10 (5.5, 10)
		
***Someone with an STI:***		
3. Would know it	5.72 (3.71)	5.5 (1, 10)
4. Could hide it	7.13 (3.31)	9 (5.5, 10)
5. Is damaged goods	3.25 (3.10)	1 (1, 5.5)
6. May not know	8.04 (2.99)	10 (6, 10)
		
***If you had an STI, you would feel:***		
7. Guilty	5.46 (3.88)	5.5 (1, 10)
8. Embarrassed	6.25 (3.86)	8 (1, 10)
		
***At the clinic:***		
9. Everyone would know if you were being tested for an STI	2.43 (2.82)	1 (1, 2)
10. Staff are discreet	9.43 (1.62)	10 (10, 10)
		
***(Only) women .......... get STIs:***		
11. Who have slept around	2.94 (3.09)	1 (1, 5.5)
12. Should know better than	4.69 (3.73)	3 (1, 9)
13. Who aren't careful	4.19 (3.58)	3 (1, 7)
		
***If someone has an STI:***		
14. People will think she is a bad person	4.27 (3.28)	3.5 (1, 6)
15. People will gossip	5.31 (3.41)	5.5 (1, 8)
16. Health workers will think poorly of her	2.86 (2.74)	1 (1, 5.5)
		
***If you had an STI:*** 17. Your partner would be angry with you	7.28 (3.47)	10 (5, 10)
		
***If someone has an STI:*** 18. People will think she is stupid	3.58 (3.04)	1.75 (1, 5.5)

Using the discrimination index, four items were flagged, including items 4 and 6 (being able to hide an STI and not knowing an STI was present), item 10 (staff discretion) and item 16 (staff morals). Using p < 0.20 as the cut-point for internal consistency, another two items were initially flagged, including item 3 (knowing an STI was present) and item 17 (partner disclosure). Internal consistency was also re-evaluated within the sub-groups emerging during exploratory analysis.

Principal component factor analysis using all items was first assessed, and the resulting eigenvalues were plotted, revealing three distinct factors arising from the item pool. Iterative factor analysis was done, omitting items one at a time. Item 4 and 17 were the first two deleted, as their factor loadings were low and both had been flagged as having low discrimination or internal consistency. Item 6 was then deleted, due to multiple factor loading, low discrimination and consistency. Finally, item 3 was dropped, as it continued to load poorly and had low internal consistency. Correlation between the final three factors (factors 1 and 2: -0.18; factors 1 and 3: 0.23; factors 2 and 3: -0.21) was not high enough to warrant combining the three sub-scales into one larger scale.

The remaining 14-items factored together in three final scales – female-specific moral stigma, social stigma and internal stigma – shown in Table 3. The four items that factored together in the first scale were derived from the work by Nack[3,4], surrounding the 'tribes of womanhood' and included moral judgement statements typically imposed on women as opposed to men, resulting in the name 'female-specific moral stigma'. All the items that factored together in the second scale referred to how the participants felt others perceived someone with an STI, prompting the name 'social stigma'. Finally, both moral and physical stigma items factored together in the third scale; however, in examining the wording of these items, all four were directed at how participants would feel about themselves if they were diagnosed with an STI, thus prompting the name 'internal stigma'.

**Table 3:** Item-total item correlation and alpha-coefficients if deleted and scale statistics for three factors identified in factor analysis

**Item**	**Factor 1:** **Female-specific Moral Stigma**		**Factor 2:** **Social Stigma**		**Factor 3:** **Internal stigma**	
	
	***r***_**(i-t)**_	***α***_**(d)**_	***r***_**(i-t)**_	***α***_**(d)**_	***r***_**(i-t)**_	***α***_**(d)**_
**1. Dirty**					0.547	0.652
**2. Violated**					0.457	0.703
**5. Damaged goods**	0.459	0.719				
**7. Guilty**					0.473	0.692
**8. Embarrassed**					0.591	0.623
9. Clinic discretion*			0.432	0.667		
10. Staff discretion*			0.413	0.673		
**11. Sleeps around**	0.598	0.632				
**12. Should know better**	0.563	0.658				
**13. Isn't careful**	0.478	0.697				
**14. Bad person**			0.398	0.676		
**15. Community gossip**			0.443	0.664		
**16. Staff morals**			0.519	0.639		
**18. Stupid**			0.404	0.675		

**N**	120		116		122	
**Alpha Co-efficient**	0.737		0.705		0.729	
**Mean**	3.77		5.16		6.59	
**SD**	2.52		1.81		2.68	

*As these items may refer to perceptions of a specific clinic, an alternative Cronbach's alpha score for the social stigma scale, eliminating these two items would be 0.647; other factors were not affected by removal of these items. Note: item 10 score was reversed for factor analysis.

In Table 3, item-total item correlations and alpha co-efficients if deleted for the three emergent factors are shown, along with scale statistics and cronbach's alpha. The alpha co-efficients for each scale were 0.737, 0.705 and 0.729 for female-specific moral stigma, social stigma and internal stigma, respectively. It is possible that two of the items (items #9 and #10) addressing women's perceptions of staff and clinic discretion may be measuring characteristics of this particular clinic, rather than of women's general perceptions, and may not be warranted in a stigma scale for wider use. Thus, an alternative analysis was run eliminating these items. The results were identical, with the exception of the social stigma scale, which now consisted of only four items and had a Cronbach's alpha score of 0.647.

Assessing each item for response and non-response characteristics, there was no large variation found; however, overall injection drug users and current sex workers were less likely to complete the stigma section of the questionnaire. As the stigma-related items were positioned at the end of the 27-item structured interview, a few women did not complete all of the questions and nine women were excluded due to incomplete answers in the stigma section of the structured interview. However, subsequent to the factor analysis and determination that the three scales did not correlate sufficiently to combine into one large scale, women who answered at least all of the stigma items represented in any one scale were included in the final calculation of Cronbach's alpha-scores shown in Table 3.

In Table 4, the associations of demographic and behavioural characteristics with the three stigma scales are shown. Higher female-specific moral stigma scores were marginally associated with being over 30 years of age, identifying as Aboriginal, Inuit or Métis and not reporting any use of injection drugs in the past six months. Among active CSW, higher social stigma scores were associated with having been working in the sex trade for less than 10 years, while among all the women, there was a marginal association of higher social stigma among women who did not report use of any non-injection drugs in the past six months. Higher internal stigma scores were associated with not having completed high school and identifying as Aboriginal, Inuit or Métis ethnicity. In a multivariable linear regression model, Aboriginal ethnicity (β = -1.63; p = 0.002) and less than a high school education (β = -1.27; p = 0.010) remained significantly associated with internal stigma score, adjusting for age, commercial sex work and injection drug use (N = 110, R-squared = 0.180). There were no significant associations in multivariable models for either the female-specific moral stigma or social stigma scales.

**Table 4:** Characteristics associated with standardized scales for female-specific moral stigma, social stigma and internal stigma, with higher scores indicating higher stigma

		**Female-specific Moral Stigma**		**Social Stigma**		**Internal Stigma**	
	***N***	**Median**	**p-value**	**Median**	**p-value**	**Median**	**p-value**
*Demographics*							
							
**Age > 30** **≤ 30**	111 10	3.25 2.12	0.085†	4.00 3.50	0.536	5.37 6.75	0.813
**High School (HS)** **No HS**	50 73	3.37 3.25	0.928	3.37 4.00	0.464	**5.50** **7.50**	0.008*
**Aboriginial, Inuit, Metis** **Other**	49 74	4.37 3.25	0.072 †	3.50 4.12	0.340	**7.75** **5.50**	0.001*
							
*Substance Use*							
							
**Injection Drug use** **No IDU**	45 73	3.00 3.62	0.076 †	3.37 4.00	0.584	6.50 7.25	0.960
**Non-Injection Drug use** **No NIDU**	96 22	3.25 4.75	0.216	3.25 4.56	0.061 †	7.19 5.50	0.346
**High Alcohol** (> 6 drinks, >1 a week) **Low Alcohol**	11 109	3.62 4.00	0.916	4.75 3.50	0.743	7.25 8.87	0.150
							
*Sexual Behaviour*							
							
**Any partner** **No partner**	96 27	3.25 3.25	0.435	3.75 4.00	0.628	6.69 5.62	0.643
**Regular partner** **No regular partner**	72 24	3.25 3.25	0.570	4.25 3.25	0.164	7.37 5.50	0.292
**Casual partner** **No casual partner**	25 71	3.25 3.25	0.418	3.25 4.00	0.773	6.00 6.62	0.880
**> 1 partner (non-client)** **≤ 1 partner**	25 69	3.37 3.25	0.185	4.00 4.00	0.614	7.12 6.25	0.702
**CSW – Never** **Former** **Current**	38 33 46	3.37 3.00 3.25	0.816	4.31 3.25 3.50	0.178	5.56 7.37 6.69	0.841
							
***Among active CSW:***							
							
**Years working** **≥ 10 years** **<10 years**	29 19	3.00 3.25	0.864	**3.25** **5.00**	0.007*	6.62 6.75	0.891
**Clients (past 6 mos)**							
**≥ 50 clients** **< 50 clients**	33 13	2.25 3.25	0.592	3.25 4.00	0.427	5.50 7.50	0.611
							
***STI Communication***							
							
**With Partner – Yes**	91	3.25	0.275	3.50	0.467	6.50	0.485
**No**	31	4.37		4.00		6.62	
**With Friend – Yes**	74	3.25	0.926	3.37	0.594	6.00	0.497
**No**	48	3.37		4.37		7.00	
**With HC worker – Yes**	110	3.25	0.640	4.00	0.705	6.66	0.966
**No**	12	2.50		3.25		6.12	

*p < 0.05; † p < 0.10

## Discussion and Conclusion

While qualitative studies can help us develop a deeper understanding of women's experiences of stigma; quantitative measures can be useful for comparing the magnitude and prevalence of stigma-related experiences across or within populations. In this paper, three distinct STI-related stigma scales emerged from a pool of items addressing STI issues including physical stigma, moral stigma, judgment by community and by healthcare workers in particular, as well as female-specific sexual categorizations of 'good' and 'bad' girls. The resulting female-specific moral stigma scale was not found to be significantly different among participants; however, there were marginally higher scores among Aboriginal women, IDUs and women with lower education levels. Social stigma was found to be higher among more recent initiates into the sex trade, while internal stigma was higher among those with lower education as well as among women reporting Aboriginal, Inuit or Métis ethnicity.

The perception of any stigma will invariably be affected by both the previous experiences of an individual as well as their current situation. Nack (2002) found that women who had higher perceptions of stigma prior to their own experiences with STIs (being tested and/or testing positive) and those women who identified as 'good' girls were more affected by the notion of being identified as a 'bad girl' [3]. Others who had previously received education about STIs as well as those who felt they already belonged in the 'bad' girl category were less concerned with these female-specific moral categories and social forms of stigma. In our study, while female-specific moral and social stigma did not vary significantly by demographic or behavioural characteristics, those with higher female-specific moral stigma scores were less likely to be active injection drug users.

In contrast, those women in our study with higher social stigma scores were less likely to be active non-injection drug users, and, among active sex workers, were more likely to have been working for less than 10 years. This may reflect an 'accommodating' phenomenon whereby women who already view themselves as 'bad' girls (i.e. active drug users, highly active sex workers) are less concerned with societal views and stigma. Societal norms, which are intrinsically tied to the social constructions of stigma, thus play a powerful role in self-identity and categorization into these predefined groups. The impact is even greater among marginalised communities, who are already separated from 'mainstream' society, generally due to a combination of factors including poverty, ethnicity, or other non-conforming behaviours (substance use, sex work). Within a marginalised community, the concept of female-specific moral stigma may serve to deepen the divide between women who are identified or self-identify as 'bad' girls, and those who self-identify as 'good' girls but who feel potentially labelled incorrectly because of their affiliation with the larger community. This may engender an intensified stigma between these groups in an effort to explicitly remove the 'community' level stigma from individual 'good' girls within the community.

High levels of female-specific moral stigma and internal stigma were found among Aboriginal women in our study. Given that minority women have historically been singled out as 'vectors and vessels' of STI transmission, the higher STI-stigma scores among Aboriginal women may represent a compounding of cultural stigma felt by these women [24-26]. And, while public health and research efforts (e.g., targeted testing and treatment of Aboriginal women in the community; numerous studies that report very high rates of HIV and STIs in this population) are meant to assist this population in accessing testing, it is possible that Aboriginal women in our study feel 'culturally targeted' – resulting in high levels of stigma and STI-stigma in particular.

Aside from this, there are other cultural factors which may be influencing the way in which the stigma scale is interpreted. The traditional meanings of female sexual morality in Aboriginal cultures may shape the way these specific stigma items, most which reflect a Western definition of feminine morality rooted in Christianity, are felt. For example, in many Aboriginal cultures, sex and sexuality are taught as being a gift, although a powerful one which needs to be respected[27]. The central idea of balance and the holistic views of health are also important and theoretically cultivate a healthy view of sexuality, as opposed to Western views which tend to stress the association between sexuality and sin[27]. In addition, in matrilineal clans female gender did not reflect lower status, and many ancient stories venerated strong, powerful female figures[28]. Despite these traditional meanings, the rise of residential schools which removed Aboriginal children from their families in order to 'educate' them in Western and Christian ways, introduced, or forced, these ways of thinking onto their existing cultures. This, coupled with Western perceptions of Aboriginal women as either "the glorified 'princess' or the denigrated 'squaw"'[28], make it difficult to predict how this entangled history may influence the present interpretation of stigma items. Nevertheless, the increased internal stigma seen in this study among Aboriginal women remained in a regression model controlling for age, education, injection drug use and sex work status indicating that a culturally-specific influence was present.

Unfortunately, it is difficult to tease out the historically gendered and socially moral underpinnings of sexuality from sexual and sexual health education. We may be more aware today of the double-standards that exist in society's view of how men and women *should *behave sexually; however, there remains an ingrained social double-standard which can, consciously or subconsciously, alter one's perceptions even among those who do not prescribe to these views. An example of this was reported by Nack (2000), where women diagnosed with an STI described their diagnosis as either 'deserved' or 'undeserved' based on their perceptions of what society viewed as acceptable behaviour and how this fit in with their own past histories[4]. In the present study, the items making up the female-specific moral stigma scale included statements with a moral tone placing the responsibility, or insinuating the fault, of STIs on women. Although the responses were skewed towards low stigma levels, a quarter of the women had moderate to high overall agreement with these items.

There are several limitations to this study. First, as the stigma scales were developed as a preliminary analysis of perceptions in this population of women, there was no inclusion of an external tool for validation. In this particular setting, it was felt that including several scales for this purpose would unduly increase respondent burden. Second, the sample was a convenience sample of women already connected with community services – therefore, we cannot generalize findings to the broader community of women in this area who may not be accessing services in any capacity. In addition, the scale categories (female-specific moral stigma, internal stigma, social stigma) that emerged from this exploratory analysis should be tested in a larger population using an hypothesis driven factor analysis (i.e. comparing the pre-supposed categories to the data). Thus, the present scales, while providing valuable insight, should undergo further development and testing to assess its usefulness on a larger scale. This is also true as the study sample size presents a limitation with respect to the ability to detect differences of less than 2.0 units in the scale scores. It is difficult to say whether the marginal results seen with differences of only 1.0 unit would become significant in a larger sample; however, the potential implications of these differences are presented and discussed as theories requiring further evaluation.

With few significant differences in stigma scores among sub-groups of women, the question of the practical use of such scales arises. Comparisons beyond demographics and behaviors, such as contact with health care providers, access of testing and treatment programs would provide a better picture of how these stigma measures may influence sexual health. Further, although the findings presented here lacked sufficient power to detect smaller differences, the suggested increased levels of female-specific moral stigma, social stigma and internal stigma among sub-groups of women is theoretically plausible and should be further evaluated in a larger population. As mentioned above, the presence of cultural differences indicates the necessary evaluation of the potentially different meanings and interpretations of STI-stigma, female sexual morals, and social perceptions held by different cultures. Importantly, the interpretations given here can only be seen as suppositions, and are limited by the lack of a priori investigation into these cultural influences and meanings.

A final limitation is that the current study only sampled women. While we developed our female-specific items from an extensive literature outlining the unique sexual categorization of women, we did not directly compare the endorsement of the items among women to similar items among men. Of note, STIs and barriers to care, including STI-related stigma, are just as important among men, and while the measure may be very different, scales that assess these experiences among men should also be developed.

The study was nevertheless able to sample a diverse group of women from a high-risk neighbourhood. In addition, while the sample was recruited from a clinic site, the evening program serves as a drop in for many services, including dinner, clothing and other aspects not directly related to seeing a health care professional. Despite the neutral setting of the evening, it *is *a clinic and, even within the context of a safe evening for women, perceptions of STI-related stigma and moral categories of 'bad' versus 'good' girls were recorded.

As public health practitioners continue to work to prevent and reduce the impact of STIs in highly marginalised populations, it will be important to consider the roles of STI-related stigma. Prevention or screening programs that are 'tailored' (rather than 'targeted') for particular high-risk groups may be less likely to add to perceptions of discrimination or stigma. In addition, in highly marginalised communities, where the population at risk represents a broad group of women, the preconceived notions of 'bad girl' membership may supersede participation in otherwise beneficial programs (e.g., a more general women's program may draw participation from a certain group of women, such as non-sex workers, creating an environment that is less desirable for another group, such as active sex workers). Thus, in the creation of new sexual health interventions and prevention messages, particular attention should be paid to the language used and the potentially subtle ways in which programs may isolate rather than integrate members of the community. For example, messages that highlight good sexual health practices and the benefits of treatment, rather than those that advertise the consequences of bad behaviour (and thereby condemn those that practice them) could help encourage individuals to seek sexual health care instead of avoiding testing for fear of a potentially stigmatizing label. In addition, efforts to assess the importance of stigma as a barrier to sexual health care should attempt to incorporate these broader aspects of the stigma experience. Sexual stigma is a deeply rooted construct in our society; however, this should not be seen as an insurmountable barrier to creating programs and policies that work to change the damaging and disproportionate impact of STI-related stigma on women.

## Competing interests

Drs. Rusch, Burgess and Stancer were involved with the weekly women's clinic program. Drs. Burgess and Stancer work at the clinic, and provide medical care during the evening program. Dr. Rusch volunteered, helping to co-ordinate activities. The doctors and clinic staff involved with the women's program did not actively participate in recruitment or data collection and women were informed that participation or refusal of participation in this study would in no way affect their normal standard of care at the clinic, nor their regular participation in the woman's program. This research was carried out as part of Dr. Rusch's graduate thesis work. The other authors have no competing interests to declare.

## Authors' contributions

MLAR, JAS, DMP and MWT were involved in the conecptualization of the study. All authors were involved in the development of the survey items. MLAR was responsible for the analysis and writing of the manuscript. All authors contributed to the editing and gave final approval on the manuscript.

## Pre-publication history

The pre-publication history for this paper can be accessed here:


